# Transcriptomic Signatures of Immune Suppression and Cellular Dysfunction Distinguish Latent from Transcriptionally Active HIV-1 Infection in Dendritic Cells

**DOI:** 10.3390/ijms27020844

**Published:** 2026-01-14

**Authors:** Shirley Man, Jade Jansen, Neeltje A. Kootstra, Teunis B. H. Geijtenbeek

**Affiliations:** 1 Department of Experimental Immunology, Amsterdam UMC Location University of Amsterdam, Meibergdreef 9, 1105 AZ Amsterdam, The Netherlands; 2 Amsterdam institute for Immunology and Infectious diseases, 1105 AZ Amsterdam, The Netherlands

**Keywords:** dendritic cells, HIV-1 transcription, HIV-1 latency, host transcription factors, transcriptomics

## Abstract

Dendritic cells (DCs) are essential for antiviral immunity but are also susceptible to HIV-1 infection. Although sensing and restriction pathways in DCs are well described, the mechanisms underlying latent infection and its functional consequences remain unclear. In this study, we performed transcriptomic profiling of monocyte-derived DCs harboring transcriptionally active (Active-HIV) or latent HIV-1 (Latent-HIV) proviruses using a dual-reporter virus. Gene set enrichment analysis revealed suppression of metabolic and stress-modulatory programs in Active-HIV compared to unexposed DCs. In contrast, Latent-HIV showed broad downregulation of pathways, including interferon and innate responses and metabolic programs, indicating a hyporesponsive and dampened antiviral state despite the absence of differentially expressed genes (DEGs). DEG analysis of Active-HIV versus Latent-HIV showed that active transcription associates with cellular stress, cytoskeletal remodeling, and RNA processing. Functional analyses further demonstrated the activation of RNA processes, the suppression of antigen-presentation pathways, and altered membrane and cytoskeletal signaling in Active-HIV. These pathways suggest that transcriptionally active HIV-1 is linked to cellular programs supporting replication, coinciding with a metabolically strained yet immunologically engaged state that may impair antigen presentation. Conversely, latently infected DCs display a hyporesponsive state consistent with proviral silencing. This dichotomy reveals distinct mechanisms of DC dysfunction that may facilitate HIV-1 persistence and immune evasion.

## 1. Introduction

Dendritic cells (DCs) are among the first immune cells to engage pathogens at mucosal entry sites, where they play a central role in initiating antiviral immunity. Upon viral sensing through pattern recognition receptors (PRRs), DCs undergo maturation and produce type I interferons (IFN) and pro-inflammatory cytokines that activate specific B- and T-cell responses [1,2]. However, in the case of HIV-1, these responses are not readily induced.

DCs express attachment receptors, such as DC-SIGN or mannose receptors, that facilitate HIV-1 capture and infection via the HIV-1 receptors CD4 and CCR5 [3,4]. Although DCs can become infected [5], several studies have shown that DCs are less permissive to productive replication due to robust intrinsic antiviral defenses that restrict reverse transcription, integration, and transcription [6,7,8]. SAMHD1, for example, restricts reverse transcription by limiting the pool of deoxyribonucleoside triphosphates [9]. The DNA sensor cyclic GMP-AMP synthase (cGAS) recognizes reverse-transcribed dsDNA, leading to the activation of type I IFN responses and restricting HIV-1 replication [10]. Notably, transcription requires innate signaling for the elongation phase in DCs [11]. Thus, integrated proviruses can fail to transition from transcription initiation to elongation, resulting in short abortive HIV-1 transcripts [12,13]. These incomplete RNAs act as pathogen-associated molecular patterns (PAMPs), engaging cytosolic PRRs and triggering innate signaling cascades that reinforce antiviral responses and drive DC maturation [14]. However, it is unclear how DC function is affected by latent HIV-1 infection.

Despite the potent antiviral capacity of DCs, HIV-1 has evolved several mechanisms to evade sensing pathways or suppress restriction mechanisms [11,14,15,16,17]. As a result, HIV-1-exposed or -infected DCs often exhibit partial or aberrant maturation [18,19,20,21,22,23]. Instead of mounting a strong type I IFN response, these DCs typically display blunted cytokine production and reduced upregulation of costimulatory molecules, thereby impairing their ability to prime effective T-cell responses [18,19]. These immune evasive strategies allow HIV-1 to persist while undermining DC-mediated antiviral immunity.

While HIV-1 sensing and restriction in DCs are well described, the mechanisms underlying latent infection and its impact on DC function remain poorly understood. Defining these processes is essential to understanding HIV-1 immunopathogenesis and the impact of early replication events on host immunity. In this study, we aimed to dissect the molecular determinants that govern HIV-1 transcriptional states in DCs and define the host transcriptional programs associated with transcriptionally active versus latent infection. Our data suggest that DCs harboring transcriptionally active HIV-1 proviruses exhibit features of metabolic strain alongside the induction of immune pathways, whereas latent proviruses are associated with a hyporesponsive transcriptional state and reduced antiviral signatures. This study provides a framework for understanding how the transcriptional state of the HIV-1 provirus shapes DC phenotype and functionality.

## 2. Results

### 2.1. Transcriptomic Analysis of DCs Harboring Transcriptionally Active or Latent HIV-1 Provirus

**Figure 1:**
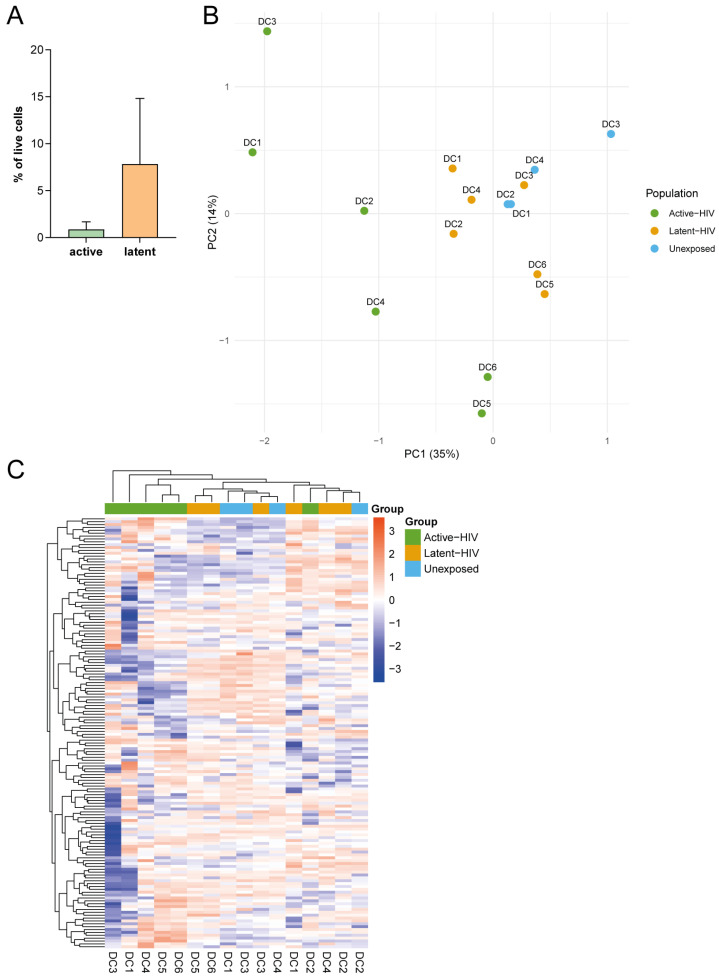
Transcriptomic clustering of isolated dendritic cells (DCs) infected with dual-reporter HIV-1. (**A**) Frequencies of primary DCs (N = 4) harboring transcriptionally active or latent HIV-1 following exposure to the dual-reporter HIV-1 as determined by flow cytometry. (**B**) Principal component analysis (PCA) plot showing the clustering of isolated primary DCs (N = 6), sorted by cell populations harboring transcriptionally active HIV-1 (Active-HIV) or latent HIV-1 (Latent-HIV). HIV-1-unexposed DCs (Unexposed) were included as controls. Donors are indicated by name and populations are indicated by color. (**C**) Hierarchical clustering of sorted samples by overall gene expression, with samples and populations indicated.

Monocyte-derived DCs from blood donors were infected with a VSV-G-pseudotyped dual fluorescence reporter HIV-1, with EF1α-driven mKO2 and HIV-1 LTR-driven GFP expression reflecting proviral integration and HIV-1 transcription, respectively [24]. Flow cytometry analyses showed distinct DC populations harboring either active (GFP+mKO2+) or latent HIV-1 provirus (GFP-mKO2+) after five days (Figure 1A). The majority of infected DCs harbored a latent HIV-1 provirus (Latent-HIV), whereas the proportion of DCs with active proviruses (Active-HIV) was low. For RNA sequencing, DCs from six donors were sorted by flow cytometry based on mKO2 and GFP expression (Supplemental Figure S1). The sorted DC populations were lysed and processed for RNA bulk sequencing. As a control, HIV-1-unexposed DCs (Unexposed) were included for RNA sequencing analysis.

After quality control and filtering of reads, the global gene expression of the samples was analyzed to determine whether there were major transcriptomic differences between donors and isolated populations. Unsupervised principal component analysis (PCA) mostly showed clustering of DCs based on HIV-1 exposure and infection state, i.e., Active-HIV or Latent-HIV, with 35% of the variance explained by principal component 1 and 14% of variance by component 2 (Figure 1B). The Latent-HIV and Unexposed DCs clustered closer together, whereas the Active-HIV populations formed a separate cluster. This indicates that clustering was not primarily based on donor variation. Hierarchical clustering of the different DC populations did not show a clear transcriptomic pattern based on HIV-1 infection status (Figure 1C). However, this unsupervised analysis showed that DCs harboring active HIV-1 provirus differed most from DCs with a latent infection and HIV-1-unexposed DCs.

### 2.2. DCs Harboring Active HIV-1 Provirus Have a More Differentiated Phenotype Compared to Latently Infected and HIV-1-Unexposed DCs

**Figure 2:**
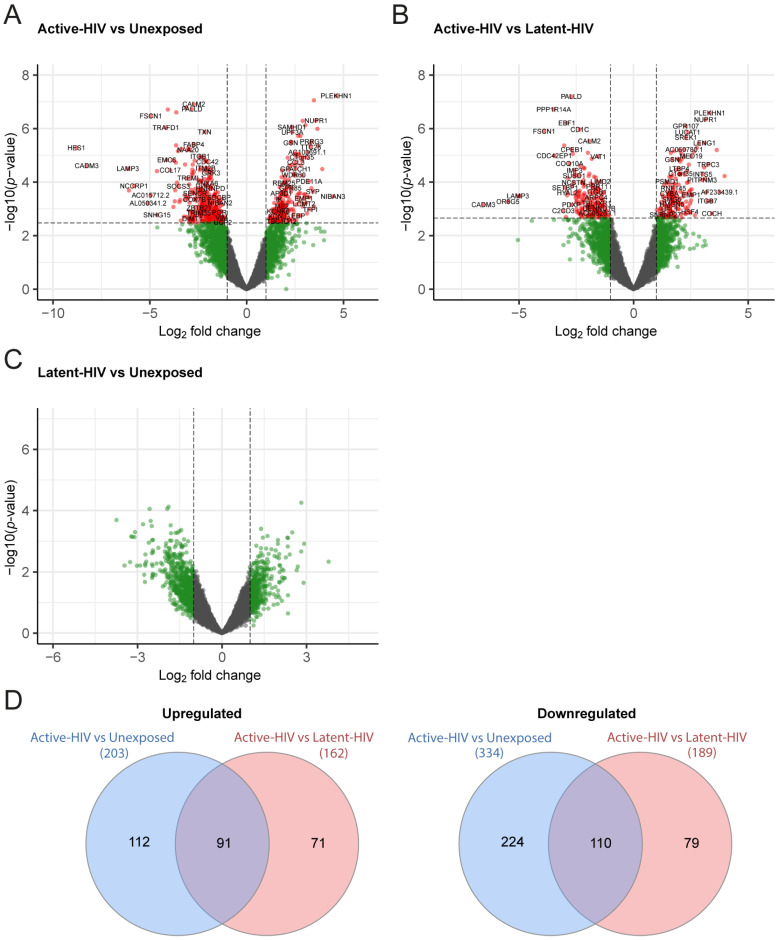
Supervised paired transcriptomic analysis of the Active-HIV, Latent-HIV, and Unexposed populations. (**A**–**C**) Volcano plots showing differentially expressed genes (DEGs) in the Active-HIV versus Unexposed (**A**), Active-HIV versus Latent-HIV (**B**), and Latent-HIV versus Unexposed comparisons (**C**). DEGs are indicated in red with gene names shown (cutoff abs log2FC > 1, adjusted *p* < 0.05). (**D**) Venn diagrams showing overlap of upregulated (**left**) and downregulated DEGs (**right**) from Active-HIV versus Unexposed and Active-HIV versus Latent-HIV populations, with the number of DEGs indicated.

Next, we analyzed the gene expression profiles of the different populations using a paired supervised differential gene expression analysis (DGEA), accounting for donor variation. A total of 537 differentially expressed genes (DEGs; abs log2FC > 1, adjusted *p* < 0.05) were found when comparing Active-HIV and Unexposed, of which 203 were upregulated and 334 were downregulated (Figure 2A and Supplemental Table S1). In contrast, 351 DEGs (162 upregulated, 189 downregulated), were found in Active-HIV versus Latent-HIV (Figure 2B and Supplemental Table S2), while no DEGs were detected for Latent-HIV versus Unexposed (Figure 2C). The Active-HIV versus Latent-HIV contrast revealed more modest transcriptional differences than the Active-HIV versus Unexposed comparison, with overall weaker fold changes. A shared subset of 91 upregulated and 110 downregulated DEGs was present in Active-HIV compared to Latent-HIV and Unexposed comparisons (Figure 2D), indicative of a core gene signature associated with transcriptional proviral activity. A total of 112 up- and 224 downregulated genes were unique to Active-HIV versus Unexposed, whereas only 71 up- and 79 downregulated genes were unique to Active-HIV versus Latent-HIV. Together, these data indicate that active HIV-1 transcription is associated with a transcriptional profile distinct from latently infected or HIV-1-unexposed DCs.

### 2.3. DCs with Transcriptionally Active Provirus Show a Functionally Impaired Phenotype, While Those with Latent HIV-1 Exhibit an Immune-Suppressed Phenotype

**Figure 3:**
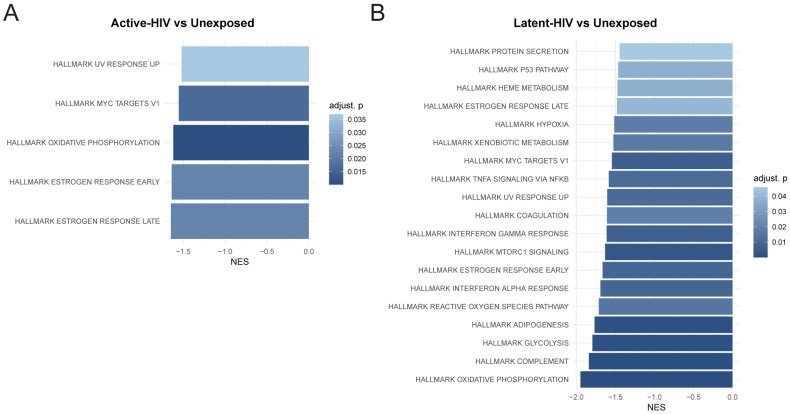
Broad functional analysis of the gene profiles of Active-HIV, Latent-HIV, and Unexposed populations. Gene set enrichment analysis (GSEA) results for the Active-HIV versus Unexposed (**A**) and Latent-HIV versus Unexposed comparisons (**B**) using the Hallmark curated gene sets. Significantly enriched pathways are shown (adjusted *p* < 0.05). NES, normalized enrichment scores.

Hallmark gene set enrichment analysis (GSEA) of Active-HIV versus Unexposed revealed significant negative enrichment of MYC targets, oxidative phosphorylation (OXPHOS), and both early and late estrogen-response pathways (Figure 3A). Reduced MYC signaling and diminished OXPHOS suggest an altered bio-energetical state of DCs harboring transcriptionally active HIV-1 [25]. The suppression of estrogen-response gene sets, which include regulators of cellular survival and immune responsiveness [26,27], may further reflect a reduced capacity of DCs with transcriptionally active provirus to maintain normal stress and immune-modulatory responses.

Notably, GSEA between Latent-HIV and Unexposed revealed many different pathways that were negatively enriched in Latent-HIV (Figure 3B). The collective suppression of IFN pathways, metabolic and bioenergetic programs, complement/coagulation cascades, TNF/NF-κB signaling, and MYC/mTOR networks indicates that latently infected DCs reside in a hyporesponsive state. While this could reflect pathway suppression induced by latent HIV-1, it is also possible that HIV-1 replication is limited in a dampened cellular state, which thereby promotes and maintains latency. Notably, Latent-HIV state is associated with decreased antiviral immunity, suggesting that heightened antiviral activity is unlikely to drive the establishment of latency. No significant Hallmark pathways were identified between Active-HIV and Latent-HIV. Thus, DCs with active HIV-1 proviruses exhibit a strained metabolic and functionally impaired state, whereas DCs with latent HIV-1 proviruses display a hyporesponsive state with suppressed pathways in immune activation, antiviral immunity, and metabolism when compared to unexposed DCs.

### 2.4. Active, but Not Latent, HIV-1 Is Associated with DC Dysfunction via Stress Signaling, Cytoskeletal Remodeling, and RNA-Processing Demands

**Figure 4:**
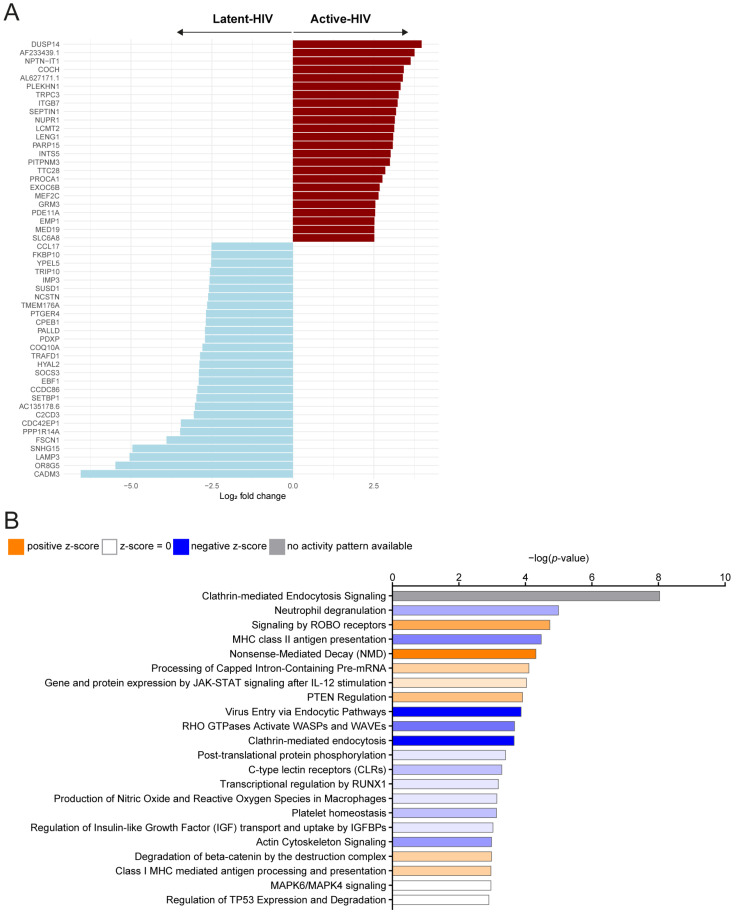
Functional analysis of the differentially expressed genes (DEGs) of Active-HIV versus Latent-HIV. (**A**) Bar plot showing the top DEGs (abs log2FC > 2.5) for Active-HIV versus Latent-HIV. Up- and downregulated DEGs for Active-HIV are indicated by color. (**B**) Overrepresentation analysis (ORA) of DEGS between Active-HIV and Latent-HIV using Ingenuity Pathway Analysis (IPA). Pathways with an adjusted *p* < 0.05 are shown. Predicted activation or inhibition is indicated by IPA Z-scores and corresponding color coding.

As GSEA using Hallmark gene sets did not reveal significantly enriched processes for Active-HIV versus Latent-HIV, we examined transcriptomic differences through focused DEG analysis. The top upregulated genes (abs log2FC > 2.5) in Active-HIV were associated with cellular stress (NUPR1, MEF2C, DUSP14) [28,29,30], cytoskeletal organization, membrane-vesicle formation and trafficking (SEPTIN1, TRPC3, EXOC6B, TTC28, PITPNM3) [31,32,33,34,35], and RNA-processing and transcriptional machinery (INTS5, PARP15, MED19, LCMT2, PLEKHN1) [36,37,38,39,40] (Figure 4A). These genes reflect the heightened signaling activity, structural reorganization, and increased biosynthetic and trafficking demands associated with productive viral replication. Conversely, key genes required for DC maturation, antigen presentation, and migration (LAMP3, FSCN1, CDC42EP1, PPP1R14A, PALLD, CADM3, CCL17) [41,42,43,44,45,46,47] were strongly downregulated in Active-HIV, along with negative regulators of inflammatory signaling (SOCS3, TRAFD1) [48,49], metabolic and mitochondrial components (COQ10A, PDXP), and RNA-binding and translation factors (CPEB1, IMP3, FKBP10) [50,51,52] (Figure 4A). This gene pattern indicates impaired antigen-presenting capacity, destabilized cytoskeletal architecture, weakened immune regulation, and reduced metabolic resilience in cells with transcriptionally active HIV-1.

Ingenuity Pathway Analysis (IPA) identified several pathways that were significantly enriched for these DEGs involved in endocytosis, cytoskeletal remodeling, RNA processing, and antigen presentation (Figure 4B and Supplemental Table S3). Clathrin-mediated endocytosis, signaling by ROBO receptors, Rho GTPase-driven actin cytoskeleton signaling, and virus-entry pathways were among the most significant, which is consistent with the extensive trafficking and structural reorganization required during productive HIV-1 infection [53,54]. Pathways associated with nonsense-mediated decay (NMD) and capped intron-containing mRNA processing further indicate increased demands on RNA metabolism during active viral transcription. Enrichment of MHC class II antigen presentation and C-type lectin receptor pathways may reflect engagement of antigen-processing machinery, while changes in neutrophil degranulation, PTEN regulation, and JAK-STAT signaling suggest broader effects on inflammatory and immune-regulatory networks.

Overall, these findings reveal that transcriptionally active HIV-1 infection of DCs is associated with a highly stressed and structurally reorganized cellular state, characterized by increased demands on RNA processing and the simultaneous suppression of core features of DC maturation, such as loss of antigen presentation, cytoskeletal stability, and immune-regulatory feedback. In contrast, latently infected DCs are likely to retain a more physiologically preserved and less perturbed or responsive phenotype.

## 3. Discussion

DCs are susceptible to HIV-1 infection; however, intrinsic restriction mechanisms substantially limit viral replication and productive infection. Consequently, latent infection is likely to occur in DCs, although the functional implications of this state remain poorly understood. By using a dual-fluorescent reporter HIV-1 to distinguish transcriptionally active from latent HIV-1 proviruses, we demonstrate that HIV-1-infected DCs adopt two transcriptionally distinct cellular states. DCs with transcriptionally active proviruses showed strong gene-level changes and the suppression of metabolic and stress-response pathways. In contrast, DCs harboring latent proviruses showed no significant DEGs compared to unexposed DCs but exhibited a hyporesponsive state with broad pathway-level suppression of antiviral, inflammatory, and metabolic programs. Focused DEG comparison revealed that DCs harboring transcriptionally active proviruses presented with increased RNA processing, cytoskeletal reorganization, stress adaptation, and antigen presentation pathways. Together, these results reveal that HIV-1 proviral transcriptional status is associated with divergent immune–metabolic states and changes to cytoskeletal architecture in DCs.

Although monocyte-derived DCs can become infected by HIV-1, different restriction mechanisms exist that limit integration and viral production [11,16,55]. Using a VSV-G-pseudotyped HIV-GKO reporter virus [24], we found that the infection of immature DCs was largely latent: over 7% of cells carried integrated but transcriptionally latent HIV-1. In contrast, fewer than 1% of DCs exhibited active proviral transcription, as marked by EF1α-driven mKO2 and LTR-driven GFP expression. These data indicate that transcriptionally silent infection is established rapidly in DCs. Furthermore, this observation aligns with previous studies demonstrating that DCs restrict productive HIV-1 infection through multiple mechanisms, including SAMHD1-mediated dNTP depletion, cGAS sensing, and other intrinsic antiviral defenses [9,10,55,56]. As a consequence, productive replication is rare, and most infected DCs contain transcriptionally silent or minimally active proviruses. To achieve sufficient infection levels for reliable isolation of infected cell populations, we employed a VSV-G-pseudotyped HIV-GKO reporter virus. This approach has some limitations due to the reporter virus lacking Env and Nef. VSV-G-mediated entry and fusion might lead to different signaling events than those triggered by HIV-1 Env [11], and the absence of Nef may influence the DC phenotypes observed, as Nef has been shown to affect different functions in DCs [57]. These considerations should be taken into account when interpreting our findings.

Unsupervised transcriptional profile analyses revealed that DCs with transcriptionally active proviruses cluster separated from both DCs harboring latent HIV-1 and unexposed DCs, indicating that active HIV-1 transcription is associated with the strongest transcriptomic remodeling. DGEA confirmed this finding, as Active-HIV versus Unexposed exhibited the majority of DEGs, whereas Latent-HIV versus Unexposed showed no significant gene-level changes despite harboring integrated proviruses. A specific set of genes was identified in Active-HIV that were differentially expressed compared to Latent-HIV and Unexposed, which likely represent genes that are consistently affected by transcriptionally active HIV-1 and are indicative of a cellular state supporting HIV-1 transcription. While these analyses capture robust differences, it remains possible that bulk RNA sequencing masks heterogeneity between cells within the infected populations.

Pathway-level analyses further revealed profound differences between the two proviral states. DCs with transcriptionally active proviruses displayed strong suppression of MYC signaling, OXPHOS, and estrogen-response pathways. Several studies have reported increased OXPHOS during early HIV-1 infection in CD4^+^ T cells [58,59,60]. However, DCs are metabolically different from CD4^+^ T cells and switch to glycolysis rather than OXPHOS upon activation [61,62]. This metabolic shift is further shaped by type I IFN signaling, which promotes glycolysis during DC maturation [63], providing a mechanism linking innate antiviral responses to the metabolic phenotype observed in DCs harboring transcriptionally active HIV-1. The decreased OXPHOS observed here is therefore consistent with the increased energetic burden of responding to virus production and innate immune activation, with prolonged stress and viral burden driving metabolic exhaustion. Moreover, the suppression of MYC signaling is consistent with reduced OXPHOS [25,64], and likely reflects the combined ER stress, innate immune activation, and mitochondrial dysfunction triggered by productive HIV-1 replication. More recently, HIV-1 infection was found to strongly downregulate MYC and downstream targets as part of transcriptomic remodeling [65], in line with our finding that MYC-dependent programs are suppressed in DCs with transcriptionally active HIV-1.

In contrast, DCs harboring latent proviruses displayed a markedly different phenotype: despite the absence of significant DEGs relative to unexposed DCs, they showed broad suppression of twenty Hallmark pathways. These included reduced IFN responses, TNF/NF-κB signaling, complement and coagulation pathways, and metabolic programs. This pattern is consistent with a coordinated but modest transcriptional shift toward a hyporesponsive, immunologically quiescent state. Such a state may be permissive for latency, both by limiting antiviral signaling or by reflecting pre-existing cellular features that predispose certain DCs to support proviral quiescence. Interestingly, the reduced antiviral pathway activity suggests that strong antiviral pressure is not a dominant feature of latency in DCs under these conditions and that DCs harboring latent provirus may instead occupy a more inert or tolerized state. Their subdued antiviral state may contribute to the maintenance of latency by reducing immune visibility; with impaired sensing of danger signals and inflammatory cues, these cells are less likely to activate surrounding immune cells or initiate antiviral responses. Whether this cellular phenotype reflects a pre-existing state that favors latency or actively promotes remains unclear.

Direct comparison of Active-HIV versus Latent-HIV DCs revealed a recurrent enrichment of pathways related to RNA processing. Notably, NMD emerged as one of the key pathways activated in DCs harboring transcriptionally active HIV-1 proviruses. NMD is a conserved mRNA surveillance system that targets aberrant or prematurely terminated transcripts for degradation, but accumulating evidence shows that HIV-1 manipulates this pathway during productive infection [66,67]. Our findings position NMD activation as a characteristic of DCs that supports active HIV-1 transcription, reflecting both antiviral surveillance and virus-driven remodeling of host RNA-decay machinery. In parallel, transcriptionally active HIV-1 was associated with the enrichment of membrane-trafficking and cytoskeletal-remodeling pathways. These signatures are consistent with the resulting activity of viral proteins, which rewire endocytic and trafficking pathways and remodel the cytoskeleton during productive infection [53,54]. This remodeling is further reflected in altered Rho-GTPase signaling and actin dynamics, which HIV-1 is known to exploit to facilitate entry, intracellular transport, and assembly while dampening antiviral signaling [68]. The observed activation of the JAK-STAT pathway in Active-HIV may also be particularly relevant given that JAK2/STAT3 inhibition can enhance DC activation [69], whereas JAK1 activity drives a tolerogenic program and induces regulatory T cells [70]. Thus, dysregulation of the Jak-STAT signaling axis in DCs could contribute to the altered activation and immune phenotype observed with transcriptionally active HIV-1. Consistent with these findings, DCs harboring transcriptionally active proviruses show downregulation of DEGs involved in cytoskeletal organization, antigen processing, and DC maturation. These transcriptomic changes likely impair dendrite formation, vesicle routing, and MHC class II antigen presentation, thereby diminishing DC-T cell crosstalk while promoting viral spread through infectious synapses. Thus, these gene signatures suggest that host trafficking and cytoskeletal architecture are modulated during transcriptionally active HIV-1 infection and are associated with impaired immune function. As this study relies on transcriptional inference without direct functional validation, future work will be needed to directly assess DC function and structural integrity in DCs harboring transcriptionally active or latent HIV-1.

The findings presented here have interesting implications for HIV-1 persistence. Although DCs promote HIV-1 persistence by transmission to CD4^+^ T cells, evidence suggests their potential as a persistent viral source. DCs sustain long-term productive HIV-1 infection [71], and more recently, in vivo findings have verified that lymph node DCs of individuals on antiretroviral therapy (ART) harbor inducible, replication-competent proviruses that can be reactivated by TLR7/8 stimulation [72]. This is consistent with our observations that latently infected DCs exhibit reduced IFN responses and TNF/NF-κB signaling, which may be overcome by TLR stimulation. Similar mechanisms have been described in latent CD4^+^ T cells, where reduced NF-κB activity has been implicated in proviral silencing and TLR7 activation can induce HIV-1 reactivation [73]. In addition, our data indicate that latent proviruses reside in cells characterized by reduced immune–metabolic activity, mirroring the low-activation resting phenotype seen in the major CD4^+^ T cell reservoir [74,75,76]. Together, this supports an immune-silent DC model in which HIV-1 preferentially persists and underscores the importance of understanding how such states arise to better define and target myeloid reservoir populations.

## 4. Materials and Methods

### 4.1. Primary Monocyte-Derived DCs

Peripheral blood monocytes were isolated from buffy coats of healthy blood donors (Sanquin, Amsterdam, The Netherlands) by Lymphoprep (Axis-Shield, Dundee, UK) density gradient centrifugation, followed by Percoll (Cytiva, Delaware, DE, USA) gradient steps. Monocytes were differentiated into monocyte-derived DCs in the presence of IL-4 (500 U/mL; Invitrogen, Carlsbad, CA, USA) and GM-CSF (800 U/mL; Invitrogen) for 6 days in RPMI supplemented with 10% FCS (Invitrogen), penicillin (10 U/mL; Invitrogen), streptomycin (10 mg/mL; Invitrogen), and L-glutamine (2 mM; Lonza, Basel, Switzerland).

### 4.2. Virus Production and Infection

The dual-reporter virus HIV-GKO [24], pseudotyped with a vesicular stomatitis virus glycoprotein (HIV-GKO_VSV-G_) and HIV-1_BAL_, were produced by co-transfection of HEK293T cells using the calcium phosphate method. Briefly, plasmid DNA was diluted in 0.042 M HEPES containing 0.15 M CaCl_2_ and carefully mixed with an equal volume of 2× HEPES-buffered saline. After 15 min, the mixture was added dropwise to HEK293T cells followed by overnight incubation at 37 °C in a humidified 3% CO_2_ incubator. The next day, medium was refreshed and HEK293T cell cultures were continued at 10% CO_2_ at 37 °C. The virus was harvested at days 2 and 3 after transfection, passed through a 0.22 µm filter, and stored in aliquots at −80 °C for later use. Virus titers were quantified by determining the TCID50 in TZM-BL cells. Before use, virus stocks were treated with DNase (Promega, Madison, WI, USA) for 30 min at 37 °C to eliminate residual plasmid DNA. DCs were infected with HIV-GKO_VSV-G_ (MOI 0.3), and after 5 days, viral infection and transcriptional activity were determined by flow cytometry.

### 4.3. Flow Cytometry and Cell Sorting

Cells were washed with phosphate-buffered saline (PBS, Thermo Fisher Scientific, Waltham, MA, USA) and stained with a fixable live/dead marker (Invitrogen, Carlsbad, CA, USA) for 30 min at 4 °C in the dark. Cells were washed twice with PBS and fixed using BD CellFIX (BD Biosciences, Franklin Lakes, NJ, USA). Samples were acquired on the BD LSRFortessa (BD Biosciences, Franklin Lakes, NJ, USA), and the data was analyzed using FlowJo software version 10 (TreeStar, Ashland, OR, USA). Cells were sorted (max. 100,000 cells/aliquot) in PBS + 10% FCS using the FACSAria device (BD Biosciences). After sorting, cells were resuspended in buffer PKD (Qiagen, Hilden, Germany) and incubated with proteinase K (0.1 μg/sample; Merck, Rahway, NJ, USA) for 1 h at 50 °C. Cells were then lysed using TriPure™ Isolation Reagent (Roche, Basel, Switzerland), and samples were stored at −80 °C until further processing.

### 4.4. RNA Sequencing and Preprocessing of Sequenced Data

Samples were processed for bulk RNA sequencing using the RNA Sequencing PLUS workflow by Single Cell Discoveries (Utrecht, The Netherlands). In brief, total RNA was processed for library preparation incorporating CEL-Seq2-style barcodes. Following RNA extraction, samples underwent concentration normalization for preliminary quality control and downstream processing. Normalized RNA was assessed on an Agilent TapeStation (Agilent, Santa Clara, CA, USA) to evaluate RNA integrity. Libraries were sequenced to a depth of approximately 20 million raw reads per sample using single-end 75 bp (1 × 75) chemistry on an Illumina NovaSeq X Plus (Illumina, San Diego, CA, USA). Raw sequencing reads were mapped to the human reference genome (GRCh38, Ensembl 98) using STARsolo (v2.7.10b). Reads that did not align to the human genome, including viral reads, were retained as unmapped and excluded from downstream analyses.

Lowly expressed genes were filtered using the filterByExpr function, and libraries were normalized using the EdgeR package (v4.8.2) [77] in R (v.4.5.0). The dataset generated in this study has been deposited at Gene Expression Omnibus (GEO), with the accession number GSE316074.

### 4.5. Transcriptomic Cluster and Differential Gene Expression Analyses

PCA was performed using EdgeR and visualized using ggplot (v4.0.1) [78], and hierarchical clustering was performed using pheatmap (v1.0.13) [79] for exploratory data analysis. Paired analyses were subsequently performed to adjust for donor-specific differences. DGEA was performed using EdgeR. *p*-values were corrected for multiple testing using the Benjamini–Hochberg method, and genes with an absolute log2FC > 1 and adjusted *p*-value < 0.05 were considered DEGs. This threshold was defined a priori to control for multiple testing and focus on transcriptional changes of likely biological relevance. The EnhancedVolcano package (v1.28.2) was used to visualize the results [80]. Venn diagrams were created using the web-based tool “InteractiVenn” [81].

### 4.6. Functional Pathway Analysis

Genes were ranked by −log(raw *p* value) × sign(log2FC), and the resulting gene rank was used for GSEA with the Hallmark curated gene sets from the Human Molecular Signatures Database (MSigDB). The Benjamini–Hochberg correction was applied for multiple testing, and pathways with an adjusted *p*-value < 0.05 were considered significant. The R packages used were msigdbr (v25.1.1), ClusterProfiler (v4.18.4), and enrichplot (v1.30.4) [82,83,84]. ORA of the DEGs was performed using the Ingenuity Pathway Analysis software (fall release 2025, Qiagen).

### 4.7. Statistical Analysis

Further data analysis and visualization was performed using GraphPad Prism software version 10 (Graphpad software Inc., San Diego, CA, USA).

## 5. Conclusions

Collectively, our data demonstrate that HIV-1 proviral transcriptional activity in DC is associated with distinct cellular states with opposing functional consequences. DCs with transcriptionally active proviruses adopt a stress-responsive, metabolically strained, and functionally impaired phenotype characterized by extensive cytoskeletal and trafficking reorganization, and diminished antigen-presenting capacity. In contrast, latently infected DCs maintain a hyporesponsive, metabolically quiescent profile with broad downregulation of antiviral and inflammatory pathways, consistent with a cellular environment that is permissive for proviral silencing. These findings suggest that HIV-1 is associated with alterations in metabolic and immune regulatory networks that modulate innate sensing, facilitate viral replication, and contribute to the establishment and maintenance of latency. Whereas transcriptionally active HIV-1 infection compromises the ability of DCs to coordinate adaptive immunity, preserved structural integrity coupled with immune quiescence may enable latently infected cells to persist. By defining the cellular programs associated with transcriptionally active versus latent HIV-1 infection, this work provides insight into how DC states shape early infection dynamics and may inform our understanding of the contribution of myeloid cells to viral persistence and immune evasion.

## Data Availability

The original data presented in the study are openly available in the GEO database at https://www.ncbi.nlm.nih.gov/geo/ with the accession number GSE316074.

## Supplementary Materials

The supporting information can be downloaded at https://www.mdpi.com/article/10.3390/ijms27020844/s1.
