# Supplementary material for: Transcriptomic Signatures of Immune Suppression and Cellular Dysfunction Distinguish Latent from Transcriptionally Active HIV-1 Infection in Dendritic Cells
Source: Int J Mol Sci. 2026 Jan 14;27(2):844. doi: 10.3390/ijms27020844 (PMC12840845; doi:10.3390/ijms27020844)

Figure S1. Gating strategy for sorting of DCs infected with dual-reporter HIV-1.

Gating strategy for sorting of different DC populations after infection with dual-reporter HIV-1. Gates are set based on HIV-1-unexposed DCs to determine autofluorescence and background signal.

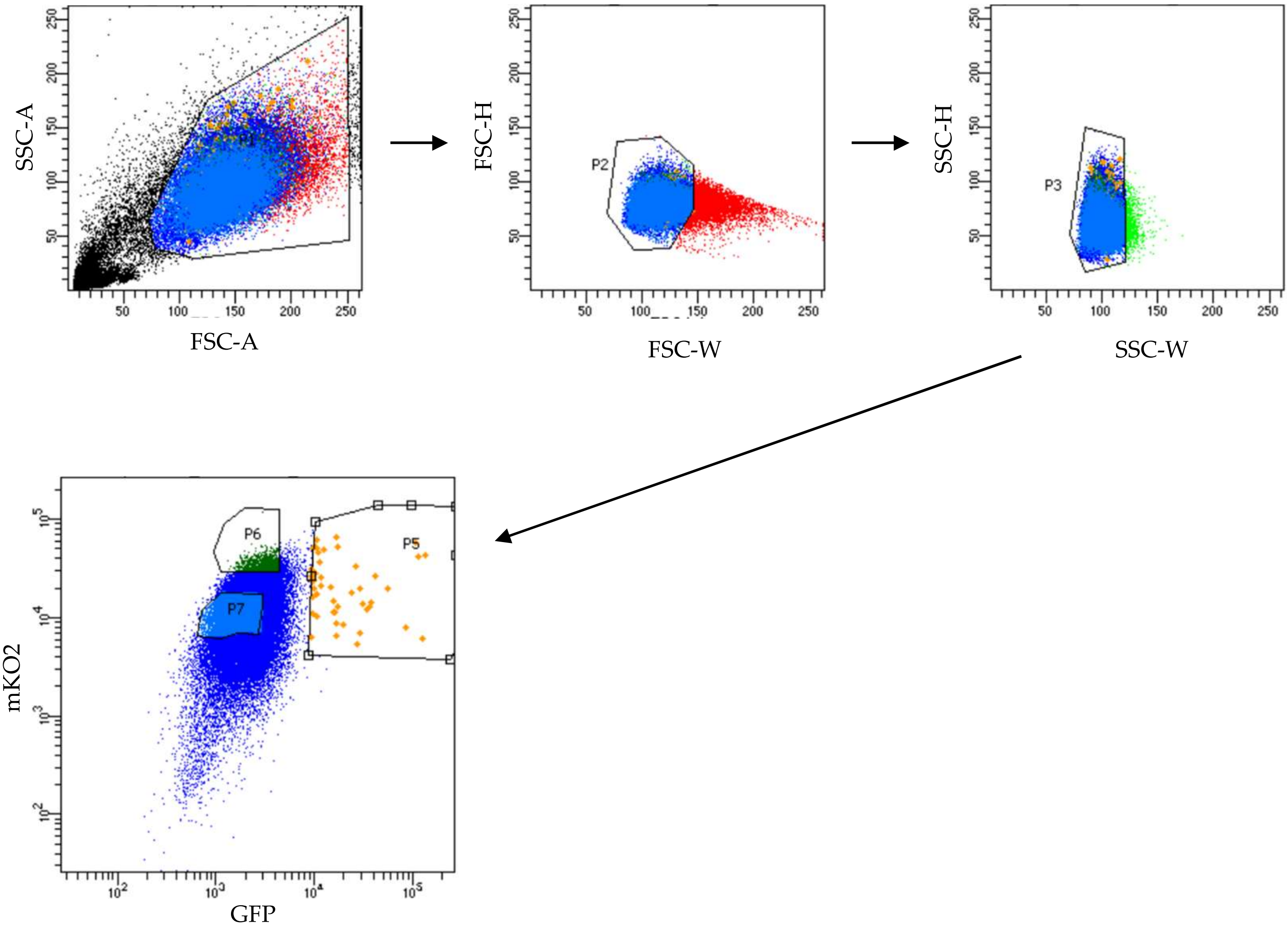

Supplement: Supplementary file 1 [file ijms-27-00844-s001.zip › Supplementary files/Figure S1.pdf]
